# Immunomodulatory and Antioxidant Activities of Select Indonesian Vegetables, Herbs, and Spices on Human Lymphocytes

**DOI:** 10.1155/2021/6340476

**Published:** 2021-03-06

**Authors:** Novi Safriani, Fransiska Zakaria Rungkat, Nancy Dewi Yuliana, Endang Prangdimurti

**Affiliations:** ^1^Department of Food Science and Technology, Faculty of Agricultural Technology, IPB University (Bogor Agricultural University), Indonesia; ^2^Department of Agricultural Product Technology, Faculty of Agriculture, Universitas Syiah Kuala, Indonesia

## Abstract

Edible plants have attracted increasing attention as functional foods as they are rich in bioactive compounds with health benefits, including antioxidant and immunomodulatory activities. However, scientific evidence of these health effects is limited. This study is aimed at determining antioxidant and immunomodulatory activities of 25 select vegetables, herbs, and spices commonly consumed in Indonesia. Phytochemical profiles were determined by measuring total flavonoid content and ^1^H-NMR. Human blood lymphocyte cells were used to probe the immunomodulatory potency and treated with the methanol extract of these vegetables, herbs, and spices. The results showed the enhanced propensity for all tested plant extracts to stimulate lymphocyte proliferation, except *Pandanus amaryllifolius*. *Etlingera elatior*, *Ocimum xcitriodorum*, *Kaempferia galanga*, and *Apium graveolens* had the highest lymphocyte cell proliferation stimulation index (SI) at concentrations of 41.67, 16.67, 4.17, and 2.5 mg/mL culture, respectively (SI 2.21 ± 0.05, 2.62 ± 0.12, 3 ± 0.05, and 2.64 ± 0.07, respectively). The NMR spectra of these four most potent plants showed low peaks in the aromatic/phenolic area and several other peaks indicating the presence of terpenoid, steroid, amino acid, and sugar compounds. The results demonstrate the immunomodulatory potential of all vegetables, herbs, and spices, except *P. amaryllifolius*, although this potential did not necessarily correlate with flavonoid content and antioxidant activity. Nevertheless, this research showed promising health effect, particularly immunomodulation, of the various local plants. Further elaboration on the specific immunomodulatory activity will be interesting.

## 1. Introduction

Plants contain various bioactive components, including alkaloids, flavonoids, coumarins, glycosides, gums, polysaccharides, phenols, tannins, terpenes, and terpenoids, which possess various biological activities that benefit health. These effects encompass antioxidant [1–3], antidiabetic [4, 5], antiobesity [6], diuretic [7], anticancer [8, 9], and anti-inflammatory [10, 11] activities, which enhance the immune system [12, 13]. The numerous studies of the benefit of food plants have resulted in the recommendation of WHO (2008) to consume 600 g of fruit and vegetables as part of a healthy diet to prevent noncommunicable diseases as well as to optimize the immune system.

Indonesia possesses numerous plant sources rich in bioactive compounds with the potential as functional foods. *K. galanga* and *C. mangga* have been known traditionally to have anti-inflammatory property and used as a daily herbal drink. *M. oleifera* has been reported to cure gout disease [14]. *A. schoenoprasum* is used to relieve pain from sunburn and sore throat [15]. Additionally, *S. androgynus*, *A. irregularis*, *M. oleifera*, *C. papaya*, *S. grandiflora*, *E. elatior*, *S. torvum*, *S. nigrum*, *T. triangulare*, *N. scutellarium*, *O. xcitriodorum*, *C. caudatus*, *P. trinervia*, *C. asiatica*, *P. indica*, *P. fruticosum*, *A. occidentale*, *C. barbata*, *A. graveolens*, *S. polyanthum*, *P. amaryllifolius*, *A. schoenoprasum*, *A. fistulosum*, *K. galanga*, and *C. mangga* have been reported to exhibit antioxidant and *α*-glucosidase inhibitory activities [16, 17]. However, research on these plants, especially on their immunomodulatory effects, has not been optimally conducted.

Flavonoids are among the most common and widely distributed phenolic components found in plant leaves, flowers, and rhizomes [1]. These compounds have been reported to have various biological activities that are beneficial to health, including antioxidant, antiallergic, anti-inflammatory, antitumor, antithrombotic, anticancer, and immunomodulatory effects [18, 19]. In addition, various effects have generally been associated with their antioxidant and metal chelating capacity [20].

Antioxidants are compounds that inhibit the oxidation process and are responsible for inhibiting the accumulation of free radicals that trigger chain reactions and damage in cells. In addition, flavonoids as antioxidants have been reported to possess the ability to inhibit LDL oxidation and confer cardioprotective effects [21]. This also reduces oxidative stress associated with atherosclerosis, cancer, aging, inflammation, and neurodegenerative diseases and functions to protect immune cells [18, 22].

Immunomodulators are substances or compounds with the tendency to either specifically or nonspecifically modulate the functionality and activity of the immune system [13, 23]. Moreover, they can also improve the proliferation of lymphocytes, which are widely used in the investigation of immune function because of their high sensitivity and enhanced propensity to propagate in culture [24].

The in vitro analysis of lymphocyte proliferation in cell culture is often conducted within a relatively short period and in easily controlled conditions [25, 26]. This study analyzed the lymphocyte proliferation activity as a preliminary screening for the immunomodulatory potential of 25 select vegetables, herbs, and spices from Indonesia. Subsequently, a correlation was established between total flavonoid content (TFC) and antioxidant activity, alongside the ability to stimulate lymphocyte cell proliferation, expressed as a stimulation index (SI) value. The samples used in this study were dried using freeze drying, and various studies have indicated that the drying method affects some of the phytochemicals, such as flavonoids, within plants. However, Mediani et al. [27] reported that freeze drying is better in order to preserve the metabolic features and beneficial values of the plants than other drying methods, including air and oven dryings.

## 2. Materials and Methods

### 2.1. Sample Preparation

Twenty-five edible plants, including vegetables, herbs, and spices (Table 1), were obtained from Bogor, West Java, Indonesia, then sorted, freeze dried, powdered, and stored in a freezer prior to analysis. Approximately 100 g of each plant was sonicated in 80% MeOH for 30 min, followed by filtration and subsequent placement in a rotary vacuum evaporator at 40°C to remove the solvent. The obtained crude extracts were stored at 4°C for further evaluation. Immunomodulatory assays were conducted using human lymphocytes with four different extract concentrations. For vegetables, the concentration was based on the WHO (2008) recommendation for daily consumption (250 g/day). In the case of herbs and spices, the concentration was calculated based on normal daily consumption (15-100 g/day and 25 g/day, respectively). These values were used to assume the quantity bioactive compounds from the plants that could be absorbed in the blood when consumed as a healthy diet and adjusted to the volume of blood in the adult human body (±6 L). This assumption was done to obtain appropriate concentrations of the plant extracts as 41.67, 16.67, 2.5, and 4.17 mg/mL culture, respectively, for the group of vegetables, two types of herbs, and spices.

### 2.2. Measurement of TFC

The TFC of samples was determined using the method of Ahn et al. [28], where 0.5 mL of 2% AlCl_3_-ethanol solution was added to 0.5 mL of plant extract, and allowed to stand for 1 h at room temperature. Measurements for absorbance were conducted with a UV-Vis spectrophotometer (Shimadzu, UVmini-1240) at a wavelength of 420 nm. These tests were repeated in triplicate, using quercetin as a reference standard. TFC was calculated in terms of milligram quercetin equivalents (QE) per gram of the dried plant material on a wet basis (mg QE/g wb).

### 2.3. Determination of Ferric Reducing Antioxidant Power

The total antioxidant potential of samples was measured using a ferric reducing antioxidant power (FRAP) assay, as described by Benzie and Strain [29]. A reagent was prepared from 2.5 mL of 10 mM TPTZ (2,4,6-tris (2-pyridyl)-1,3,5-triazine) solution in 40 mM hydrochloric acid with 2.5 mL of 20 mM ferric chloride and 25 mL of 300 mM acetate buffers at pH 3.6, and then, 3 mL of this reagent was added to 0.1 mL of plant extract. The mixtures were allowed to react for 10 min at 37°C and followed by absorbance measurement at 593 nm in triplicate. Subsequently, FRAP was calculated using an ascorbic acid calibration curve, which was expressed in milligram ascorbic acid equivalents (AAE) per gram of dried plant material on a wet basis (mg AAE/g wb).

### 2.4. Lymphocyte Cell Isolation

Lymphocytes were isolated according to the procedure that had been described by Erniati et al. [30], with modifications in the number of blood samples. Peripheral blood samples (30 mL) were aseptically collected from a healthy adult human female with the approval of the IPB University Ethical Committee. The blood was immediately transferred into sterile tubes and centrifuged at 300 × *g* at 20°C for 10 min. The buffy coat layer obtained was passed through a Ficoll-Hypaque solution (Sigma-Aldrich) and centrifuged at 900 × *g* at 20°C for 30 min to obtain a lymphocyte ring. The cells were aspired carefully, PBS (Sigma-Aldrich) was added, and the solution was centrifuged again to obtain lymphocyte cell pellets. These were washed with PBS, and the cell viability was calculated by adding 10 *μ*L of trypan blue to 10 *μ*L of the cell suspension followed by counting with a hematocytometer under a microscope. The cells were further diluted with RPMI-1640 medium (Sigma-Aldrich) containing 10% fetal bovine serum, 100 U/mL penicillin, and 100 *μ*g/mL streptomycin (Sigma-Aldrich) to obtain a density of 1 × 10^6^ cells/mL. The cell suspensions used to determine SI had viability ≥ 95%.

### 2.5. Lymphocyte Proliferation Activity Determination

**Figure 1:**
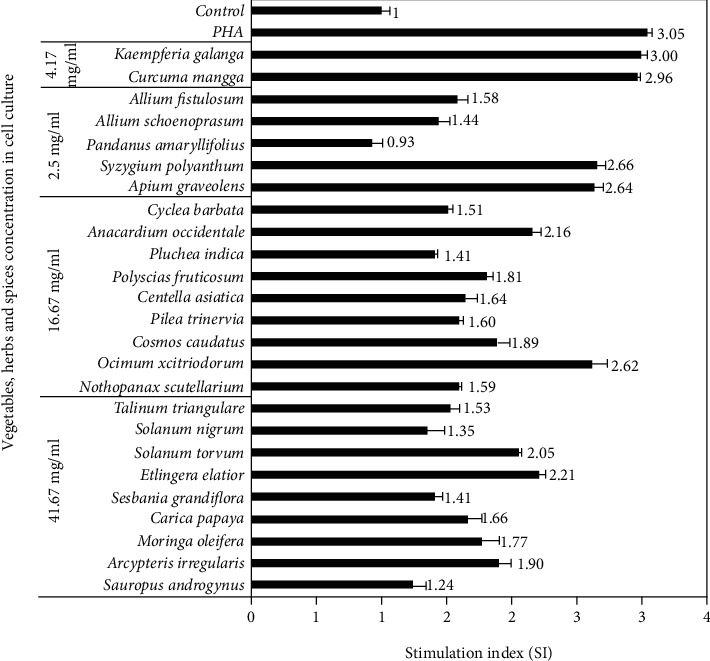
The effect of 25 select vegetable, herb, and spice extracts on human peripheral lymphocyte proliferation in vitro (*n* = 3, error bars represent standard deviation).

The lymphocyte proliferation activity test was based on the method described by Erniati et al. [30] with some modifications. A total of 80 *μ*L of lymphocyte cell suspension (1 × 10^6^ cells/mL) was added into 96-well plates, alongside 20 *μ*L of the plant extracts at various concentrations, as 41.67, 16.67, 2.5, and 4.17 mg/mL culture, respectively, for the group of vegetables, two types of herbs, and spices (Figure 1). Meanwhile, phytohemagglutinin (PHA) and RPMI media were added to other wells as positive and negative controls, respectively. All preparations were incubated at 5% CO_2_, 37°C, and 90% RH for 72 h. Four hours prior to the termination of the incubation period, 10 *μ*L 3-(4,5-dimethylthiazole-2-yl)-2,5-diphenyl tetrazolium bromide (MTT) (5 mg/mL) was added to each culture well. After incubation, ethanol was added (100 *μ*L per well), and the absorbance was measured using an ELISA reader (BioGen) at 595 nm. Furthermore, the optical density (OD) value was used to calculate the SI, indicating proliferation activity, which was calculated using the following equation:

(1) $$\begin{array}{c} \text{Stimulation index}=\frac{{\text{OD}}_{\text{treatment cell}}}{{\text{OD}}_{\text{negative control cell}}}. \end{array}$$

### 2.6. ^1^H-NMR Analysis

The chemical profile of plant extracts was determined using ^1^H-NMR as described by Wijaya et al. [31] with solvent modification. The extract samples were diluted with CD_3_OD, vortexed for 2 min at 25°C, ultrasonicated for 15 min, and centrifuged at 1000 × *g* for 15 min. The mixture was then transferred into a 5 mm NMR tube and analyzed using a 500 MHz NMR (JEOL NMR spectrometer, USA). Phasing, baseline, and reference corrections of NMR spectra were performed manually using MNOVA version 13.0. The metabolites were identified on the basis of comparison of ^1^H-NMR spectra of the samples with those of published literature.

### 2.7. Statistical Analysis

The data were reported as the mean ± standard deviation of at least triplicate determinations and then statistically analyzed using one-way ANOVA, followed by the Duncan test. Furthermore, differences were considered significant at *p* values < 0.05. The ANOVA, Duncan test, and Pearson correlation were evaluated using IBM SPSS Statistics software, version 21.

## 3. Results and Discussion

### 3.1. Total Flavonoid Content

The determination of TFC was conducted using the aluminum chloride colorimetric method (AlCl_3_ coloration), based on the complex formation between aluminum chloride and the keto groups on C-4 atoms and the hydroxy groups on C-3 or C-5 atoms present in flavones and flavonols [32]. The TFC in vegetable, herb, and spice extracts was reported in terms of QE concentration (mg/mL).

The values obtained varied from 0.825 to 24.598 mg QE/g of dried samples, as seen in Table 2. *Cosmos caudatus* had the highest TFC, followed by *Moringa oleifera* and *Pilea trinervia*, at 24.60, 15.44, and 12.33 mg QE/g, respectively, whereas *Kaempferia galanga* exhibited the lowest TFC (0.825 mg QE/g of dried sample).

The flavonoid content of *C. caudatus* (24.60 mg QE/g dried leaves, equivalent to 4.88 mg QE/g fresh leaves) recorded in this research was lower than the value reported by Sukrasno et al. [33], at 14.61 mg routine equivalents per gram fresh leaves. This is possibly influenced by variations in the analytical methods used, as Sukrasno et al. used HPLC, and the results were calculated in terms of routine equivalents. Furthermore, the main flavonoid components observed were quercetin and routine [33]. In addition to analytical methods, plant-growing areas or environments are also known to influence the content of plant metabolites [34].

*M. oleifera* was also high in flavonoid content (15.44 mg QE/g dried leaves, equivalent to 65,415 mg QE/g extract), evaluated as slightly higher than that reported by Castillo-lópez et al. [35] at 60.26 mg QE/g extract. Moreover, the main components in *M. oleifera* leaves included myricetin, quercetin, and kaempferol [36].

The total phenolic content of the vegetables, herbs, and spices used in this study was previously reported by Syabana et al. [16] and Yuliana et al. [17] as varying from 0.08 to 37.99 *μ*g gallic acid equivalent (GAE)/mg extract (Table 1). Similar to flavonoid content, *C. caudatus* was reported to have high phenolic content (21.34 *μ*g GAE/mg extract) whereas *K. galanga* exhibited low phenolic content (1.53 *μ*g GAE/mg extract). However, a different trend was observed in *M. oleifera. M. oleifera* had a high flavonoid content, but the total phenol content was low compared with that of other plants. Vegetables, herbs, and spices contain a wide variety of phenolic compounds in addition to flavonoids. Flavonoids and other phenolic compounds are known to have antioxidant activities related to protecting the lymphocyte cell membranes from free radical oxidation and stimulating cell proliferation [18, 30]. According to Kale et al. [19], these compounds play a role at the cellular level, which includes inhibiting the activation of procarcinogens and proliferation of cancer cells, ensuring selective cell death by apoptosis, preventing metastases and angiogenesis, activating immune responses, and modulating inflammatory cell cascades and drug resistance.

### 3.2. Antioxidant Activity

The antioxidant activity in 25 vegetables, herbs, and spices was determined using the FRAP method, based on the ability of the extract to reduce radical promoting ferric to ferrous ion. The plant extracts tended to donate electrons capable of this effect, in an attempt to ensure proportionality. Thus, the FRAP value of plant extracts was positively correlated with their ability to donate electrons [29].

The antioxidant activity in vegetables, herbs, and spices evaluated in this study was reported in terms of the AAE concentration (mg/g of dried sample), shown in Table 2 to range from 1.07 to 17.52 mg AAE/g. *C. caudatus* demonstrated the highest value, followed by *M. oleifera* and *Solanum torvum*, at 9.45 and 8.15 mg AAE/g, respectively. Moreover, *Allium schoenoprasum* and *K. galanga* exhibited the lowest activities at 1.07 and 1.10 mg AAE/g, respectively.

The FRAP values indicated the propensity for the plant components to donate electrons and stop chain oxidation reactions by reducing oxidized intermediate compounds to more stable forms, subsequently enhancing the potential for use as natural antioxidants [37]. Antioxidant components within plant extracts are multifunctional, and their activity and mechanism largely depend on the composition and conditions of the system. Syabana et al. [16] and Yuliana et al. [17] reported the 1,1-diphenyl-2-picrylhydrazyl (DPPH) radical scavenging activity of the vegetables, herbs, and spices used in this study, varying from 10.30% to 95.11% (Table 2). Similar to the FRAP values, *C. caudatus* demonstrated high ability to scavenge the DPPH free radical (94.68%). Meanwhile, *M. oleifera* and *S. torvum* exhibited moderate ability in scavenging the free radical (47.10% and 67.14%, respectively), whereas *K. galanga* showed low free radical scavenging activity (22.15%).

The Pearson correlation test indicated the existence of a significant positive correlation between the TFC of samples tested and antioxidant activities, via both FRAP and DPPH (*r* = 0.755, *p* < 0.01 and *r* = 0.277, *p* < 0.05, respectively). Similarly, total phenolic content had a significant positive correlation with antioxidant activity by FRAP and DPPH (*r* = 0.477 and 0.785, respectively, *p* < 0.01). These outcomes were consistent with the report by Fidrianny et al. [38], which established a significant linear correlation between both parameters in plant extracts. The activity was supported by the presence of OH functional groups, and a larger number of these groups are implicated in enhanced antioxidant activity [39]. Hence, there are indications that these functional groups possess the capacity to reduce free radicals.

### 3.3. Stimulation Index

The activity of lymphocyte cell proliferation was determined using SI value [30] via MTT staining, based on the conversion of MTT salt to purple formazan compounds. This change is due to the succinate dehydrogenase enzyme activity in the mitochondria of living cells, and the number of crystals formed, determined by the intensity of the absorbance at 570 nm, is directly proportional to the amount of living lymphocyte cells. The comparison of the SI of the 25 vegetable, herb, and spice extracts normalized to the control and mitogen (PHA) wells is shown in Figure 1.

All samples possessed some capacity to stimulate human lymphocyte proliferation in vitro, indicating immunomodulatory potential, except for *Pandanus amaryllifolius* (Figure 1). Furthermore, at 41.67 mg/mL, *Etlingera elatior* was observed to have the highest SI (2.21), followed by *S. torvum* (2.05). Among the samples added at 16.67 mg/mL, *Ocimum xcitriodorum* was the most potent (SI = 2.62), whereas *Syzygium polyanthum* and *Apium graveolens* shared high SI at 2.66 and 2.64, respectively, at a concentration of 2.5 mg/mL. Also, the addition of *K. galanga* and *Curcuma mangga* at 4.17 mg/mL exhibited high SI of 2.99 and 2.97, respectively. The values of all samples were observed to be lower than 3.05, which was the SI of the PHA-positive control. PHA is a lectin commonly found in plants, with the propensity to function as a mitogen, an agent capable of inducing cell division, in both T and B cells [13]. This is likely because the tested samples in this study were still in the form of crude extract, in which the active compounds might still have been relatively minor in proportion.

**Figure 2:**
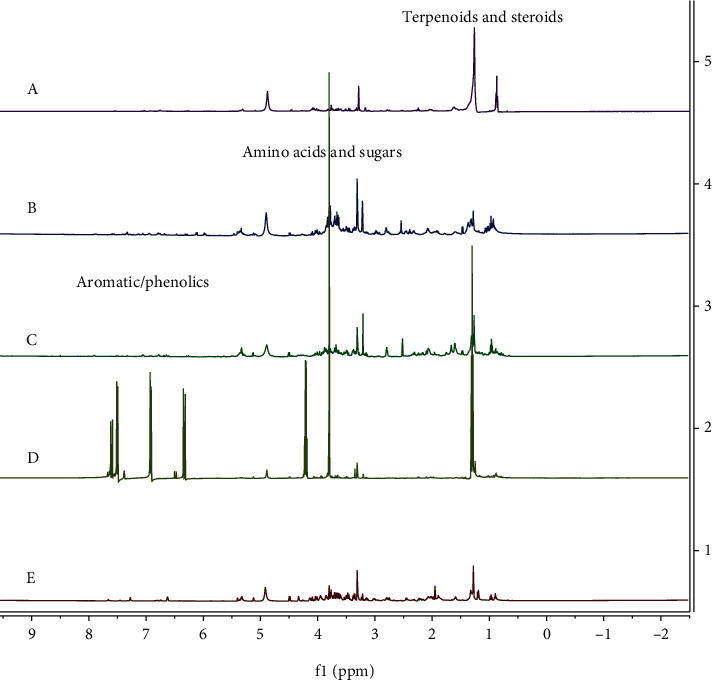
^1^H-NMR profile of *E. elatior* (a), *A. graveolens* (b), *O. xcitriodorum* (c), *K. galanga* (d), and *P. amaryllifolius* (e) extracts.

**Figure 3:**
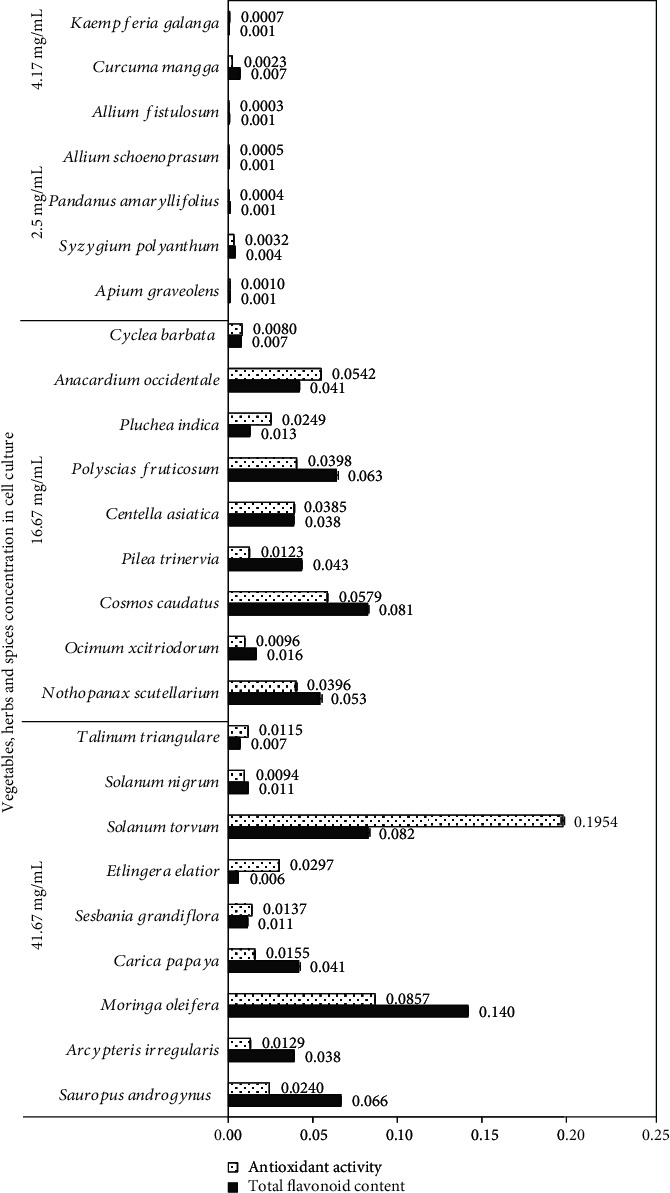
Total flavonoid content (mg QE/mL) and antioxidant activity (mg AAE/mL) of 25 select vegetables, herbs, and spices in cell culture (*n* = 3, error bars represent standard deviation).

The lymphocyte cell proliferation effect following treatment with the extracts of vegetables, herbs, and spices was assumed to be influenced by the presence of various phytochemicals present in these extracts encompassing carotenoids [40], flavonoids [18], phenolics [41], and polysaccharides [42]. Also, NMR spectrum results (Figure 2) showed the presence of various aromatics and phenolics, terpenoids and steroids, and amino acids and sugars, as indicated by chemical shifts within the ranges of 6-8, 0.5-2, and 2-6 ppm, respectively [43]. These compounds possibly played a role in the elevated activity of lymphocyte proliferation, the variations in the concentration of the extracts added to cell culture, the TFC, and the subsequent antioxidant activity in cell culture (Figure 3).

The Pearson correlation test was used to ascertain the correlation between TFC and antioxidant and immunomodulatory activities in cell culture. There was no significant correlation (*p* > 0.05) found between TFC and antioxidant activity and immunomodulatory activity, presumably because the flavonoid content was minimal in cell culture (Figure 3). However, other bioactive components that play a role in lymphocyte cell proliferation were identified in the sample extract.

A possible mechanism of lymphocyte activation by bioactive components in vegetables, herbs, and spices was assumed to be via the attachment of mitogens to the cell surface of lymphocytes, e.g., PHA. The bioactive components found in the test plants might function as mitogen ligands for receptors on the surface of T and B cells [44], subsequently activating signal transduction through second messengers including inositol triphosphate. This could further stimulate the release of Ca^2+^ into the cytoplasm, the increase in concentration of which plays an important role in stimulating the activation of protein kinase C, which can induce expression of genes such as IL-2 that leads to IL-2 production. IL-2 promotes the proliferation of B and T cells [45]. Therefore, this alleged mechanism of lymphocyte activation by mitogens in plants may be confirmed via IL-2, B cell, and T cell analyses. Nevertheless, it seems that this ligand binding activation of lymphocyte does not depend on the antioxidant capacity of the flavonoid and phenolic compounds as shown by the uncorrelated total flavonoid content and antioxidant with immunomodulatory activity in the cell culture. This assumption needs further research.

### 3.4. ^1^H-NMR Profile of Five Plant Extracts

The chemical profiles of the most promising extracts were analyzed using ^1^H-NMR. The results showed differences in chemical shifts, as most of the samples exhibited peaks in the aromatic/phenolic compound areas (*δ* 6-8 ppm) [43] with varying intensities. The profiles of the four most active plant extracts, having the highest SI values *including E. elatior*, *A. graveolens*, *O. xcitriodorum*, and *K. galanga*, are shown in Figure 2. The profile of *P. amaryllifolius* found to be inactive with the lowest SI is used as a comparison (Figure 2).

The ^1^H-NMR profile shows a similarity in the chemical shift pattern of all five extracts, possessing a peak in the aromatic/phenolic compound range (*δ* 6-8 ppm), terpenoid and steroid range (*δ* 0.5-2 ppm), and amino acid and sugar range (*δ* 2-6 ppm). Moreover, high-intensity signals were observed in the region of *δ* 3.0-5.5 (Figure 2), which could be assigned as saccharides/sugars, mainly *α*-glucose (*δ* 5.11-5.13, d; *J* = 3.8 Hz) and *β* glucose (*δ* 4.46-4.5, d; *J* = 7.8 Hz). Other high-intensity signals could also be attributed to amino acids, such as threonine (*δ* 1.32, d, *J* = 6.6 Hz), alanine (*δ* 1.45-1.48, d, *J* = 7.2 Hz), and glutamate (*δ* 4 2.04-2.07, m) [43].

Figure 2 shows the presence of higher peaks in *E. elatior*, *A. graveolens*, *O. xcitriodorum*, and *K. galanga* extracts in contrast with that of *P. amaryllifolius*. Despite the low TFC (Table 2), *K. galanga* possesses higher peaks in the aromatic/phenolic areas (*δ* 6-8 ppm) [43] compared with other plants, which presumably is due to the presence of other phenolic components than flavonoids. Phenolic compounds, encompassing flavonoids, are specifically known as natural antioxidants with a variety of biological and pharmacological activities that are beneficial to health [1].

The *K. galanga* rhizome is rich in bioactive compounds including terpenoids, diarylheptanoids, esters, phenolic glycoside, flavonoids, phenolic acids, benzoic acids, and polysaccharides [46]. It in particular is known to contain 2.5-4% of essential oil with ethyl cinnamate, trans-ethyl p-methoxycinnamate, cis-ethyl p-methoxycinnamate, p-methoxycinnamic acid, and monoterpene ketone as the main compounds, as well as other components such as borneol, 1,8-cineole, 3-carene, (E)-cinnamaldehyde, eucalyptol, kaempferol, methyl p-coumaric acid ethyl ester, pentadecane, hexadecane, and heptadecane [47].

**Figure 4:**
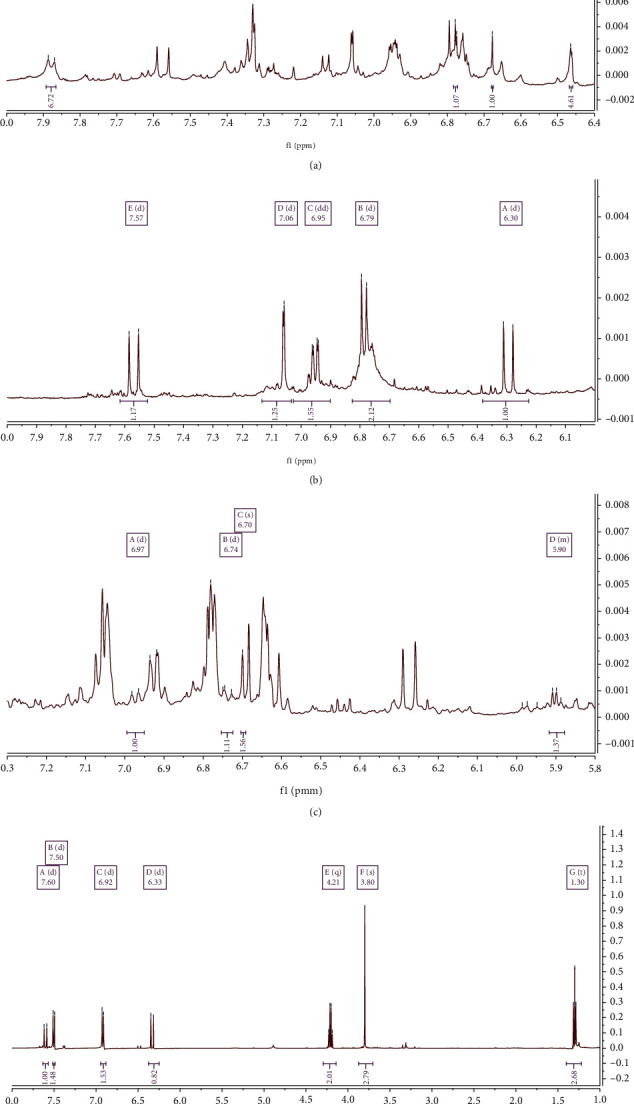
^1^H-NMR profile of *A. graveolens* with chemical shift of apiin (a), *E. elatior* with chemical shift of caffeic acid (b), *O. xcitriodorum* with chemical shift of eugenol (c), and *K. galanga* with chemical shift of ethyl p-methoxycinnamate (d).

Ethyl p-methoxycinnamate has been identified as one of the major phytochemical constituents that possess various pharmacological activities, encompassing anti-inflammatory and analgesic [48, 49], anticancer [50], antiangiogenic [47], and antimicrobial activities [51]. On the basis of the ^1^H-NMR spectrum comparison reported by Hasali et al. [52], it was found that the ethyl p-methoxycinnamate component was detected in the ^1^H-NMR spectrum of *K. galanga* extract in this study at *δ* 7.60 (d, *J* = 16.0 Hz, 1H), 7.50 (d, *J* = 8.7 Hz, 1H), 6.92 (d, *J* = 8.9 Hz, 2H), 6.33 (d, *J* = 16.0 Hz, 1H), 4.21 (q, *J* = 7.1 Hz, 2H), 3.80 (s, 3H), and 1.30 (t, *J* = 7.2 Hz, 3H) (Figure 4). Moreover, kaempferol was detected in the ^1^H-NMR spectrum at *δ* 6.91 (d, *J* = 8.8 Hz, 2H), 6.40 (d, *J* = 1.8 Hz, 1H), and 6.19 (d, *J* = 1.8 Hz, 1H), and luteolin at *δ* 6.90 (d, *J* = 8.8 Hz, 1H), 6.44 (d, *J* = 2.2 Hz, 1H), and 6.21 (d, *J* = 2.2 Hz, 1H) [53].

*E. elatior* contains various bioactive components, including flavonoids, phenolic acids, terpenoids, saponins, tannins, steroids, and carbohydrates, as well as essential oils with *α*-pinene, decanal, and 1-dodecanol [54]. In addition, the phenolic acid components include chlorogenic, ferulic, and caffeic acids [55], which have been specifically reported to possess various biological activities, encompassing its role as an antioxidant, anti-inflammatory, anticarcinogenic, and immunomodulatory agent [56, 57]. On the basis of the ^1^H-NMR spectrum reported by Silva et al. [53], caffeic acid was detected in the ^1^H-NMR spectrum of *E. elatio*r extract in this study at *δ* 7.57 (d, *J* = 15.9 Hz, 1H), 7.06 (d, *J* = 2.1 Hz, 1H), 6.95 (dd, *J* = 8.2, 2.1 Hz, 2H), 6.79 (d, *J* = 8.2 Hz, 2H), and 6.30 (d, *J* = 15.9 Hz, 1H) (Figure 4). Additionally, quercetin was detected in the ^1^H-NMR spectrum at *δ* 7.73 (d, *J* = 2.2 Hz, 1H), 7.64 (dd, *J* = 8.2, 2.2 Hz, 1H), 6.40 (d, *J* = 2.0 Hz, 1H), and 6.89 (d, *J* = 8.2 Hz, 1H) [53].

*A. graveolens* extract contains carbohydrates, phenols, flavonoids, alkaloids, steroids, glycosides, furocoumarins, volatile oils, sesquiterpene alcohols, fatty acids, and trace elements (sodium, potassium, calcium, and iron). In addition, the furocoumarins include celerin, bergapten, apiumoside, apiumetin, apigravrin, osthenol, isopimpinellin, isoimperatorin, celereoside, and 5 and 8-hydroxy methoxypsoralen, whereas the phenols consist of graveobioside A and B, apiin, apigenin, isoquercitrin, tannins, and phytic acid. Apiin is a bioactive component that has been reported to possess anti-inflammatory activity [52, 53], and the ^1^H-NMR spectrum reported by Hasali et al. [52] shows its detection in *A. graveolens* extract at *δ* 7.88 (d, *J* = 8.5 Hz, 7H), 6.78 (d, *J* = 1.7 Hz, 1H), 6.68 (s, 1H), and 6.46 (d, *J* = 2.0 Hz, 5H) (Figure 4). Apigenin was also detected in the 1H-NMR spectrum at *δ* 7.85 (d, *J* = 8.8 Hz, 2H), 6.94 (d, *J* = 8.8 Hz, 2H), 6.47 (d, *J* = 2.0 Hz, 1H), and 6.22 (d, *J* = 2.0 Hz, 1H) [53].

*O. xcitriodorum* leaves contain alkaloids, phenolic compounds, tannins, lignin, starch, saponins, flavonoids, terpenoids, and anthraquinone. Also, they are rich in essential oils that contain various chemical compounds, encompassing *γ*-terpinene, fenhone, linalool, *α*-terpineol, methyl chavicol, nerol, neral, geraniol, geranial, neryl acetate, methyl cinnamate, *β*-caryophyllene, trans-*α*-bergamotene, *α*-humulene, *α*-bisabolene, *β*-myrcene, 1,8-cineole, *α*-farnesene, eugenol, and benzoic acid [58, 59]. Eugenol has been reported to possess some pharmacological activities, including anti-inflammatory [60], antipyretic, and analgesic [61] activities. On the basis of the comparison of ^1^H-NMR spectrum in Japanese Advanced Industrial Science and Technology (AIST) [62], eugenol was detected in the ^1^H-NMR spectrum of *O. xcitriodorum* extract in this study at *δ* 6.97 (d, *J* = 8.4 Hz, 1H), 6.74 (d, *J* = 9.1 Hz, 1H), 6.70 (s, 2H), and 5.92-5.88 (m, 1H) (Figure 4).

## 4. Conclusions

The results of this research indicate the propensity for all selected vegetables, herbs, and spices to stimulate lymphocyte proliferation and demonstrate immunomodulatory potential, excepting *P. amaryllifolius*. This performance does not necessarily correlate with the flavonoid and antioxidant activities. *E. elatior* extract promoted the highest lymphocyte cell proliferation activity at a concentration of 41.67 mg/mL, followed by *O. xcitriodorum* at 16.67 mg/mL, *K. galanga* at 4.17 mg/mL, and *A. graveolens* at 2.5 mg/mL. In addition, NMR spectra of these extracts exhibited low peaks in the aromatic and phenolic areas, despite the presence of several other peaks that indicated the presence of terpenoid, steroid, amino acid, and sugar compounds. The fact that the antioxidant capacity of the flavonoids and phenolic compounds is uncorrelated with the lymphocyte's proliferation activity needs further research. In addition, further investigations are required to identify the specific compounds responsible for the cell proliferation activity and subsequently elucidate their mechanism of action.

## Figures and Tables

**Table 1:** Names and part of 25 select vegetables, herbs, and spices used.

No.	Scientific name	Local name	Part used
1	*Sauropus androgynus*	Katuk	Leaf
2	*Arcypteris irregularis*	Pakis	Leaf
3	*Moringa oleifera*	Kelor	Leaf
4	*Carica papaya*	Pepaya	Flower
5	*Sesbania grandiflora*	Turi	Flower
6	*Etlingera elatior*	Kecombrang	Flower
7	*Solanum torvum*	Takokak	Fruit
8	*Solanum nigrum*	Leunca	Fruit
9	*Talinum triangulare*	Ginseng Jawa	Leaf
10	*Nothopanax scutellarium*	Mangkokan	Leaf
11	*Ocimum xcitriodorum*	Kemangi	Leaf
12	*Cosmos caudatus*	Kenikir	Leaf
13	*Pilea trinervia*	Pohpohan	Leaf
14	*Centella asiatica*	Pegagan	Leaf
15	*Polyscias fruticosa*	Kedondong Cina	Leaf
16	*Pluchea indica*	Beluntas	Leaf
17	*Anacardium occidentale*	Jambu mete	Leaf
18	*Cyclea barbata*	Cincau	Leaf
19	*Apium graveolens*	Seledri	All parts
20	*Syzygium polyanthum*	Salam	Leaf
21	*Pandanus amaryllifolius*	Pandan	Leaf
22	*Allium schoenoprasum*	Bawang kucai	All parts
23	*Allium fistulosum*	Bawang	Leaf
24	*Curcuma mangga*	Temu mangga	Rhizome
25	*Kaempferia galanga*	Kencur	Rhizome

**Table 2:** Total flavonoid content, phenolic content, and antioxidant activities of 25 select vegetables, herbs, and spices.

No.	Vegetables, herbs, and spices	Flavonoid content	Phenolic content^∗^	Antioxidant activities	
		(mg QE/g)	(*μ*g GAE/mg extract)	FRAP (mg AAE/g)	DPPH (%)^∗^
1	*Sauropus androgynus*	6.58 ± 0.01^n^	2.10 ± 0.12^de^	2.40 ± 0.001^g^	85.60 ± 1.18^m^
2	*Arcypteris irregularis*	5.35 ± 0.04^k^	13.38 ± 0.08^k^	1.80 ± 0.012^d^	94.24 ± 0.38^n^
3	*Moringa oleifera*	15.44 ± 0.03^t^	5.27 ± 0.09^gh^	9.45 ± 0.001^u^	47.10 ± 0.50^gh^
4	*Carica papaya*	6.21 ± 0.17^m^	4.47 ± 0.10^g^	2.35 ± 0.044^f^	10.30 ± 2.05^g^
5	*Sesbania grandiflora*	2.38 ± 0.07^e^	1.07 ± 0.36^bc^	2.94 ± 0.011^h^	14.87 ± 1.18^bc^
6	*Etlingera elatior*	1.56 ± 0.01^c^	14.43 ± 0.83^l^	7.92 ± 0.020^s^	92.64 ± 1.18^l^
7	*Solanum torvum*	3.41 ± 0.04^i^	4.70 ± 0.32^g^	8.15 ± 0.043^t^	67.14 ± 1.63^g^
8	*Solanum nigrum*	2.42 ± 0.02^e^	2.46 ± 0.45^ef^	1.98 ± 0.005^e^	28.48 ± 1.20^ef^
9	*Talinum triangulare*	1.78 ± 0.05^d^	6.24 ± 0.23^ij^	3.02 ± 0.001^i^	65.41 ± 0.72^ij^
10	*Nothopanax scutellarium*	9.78 ± 0.24^q^	2.95 ± 0.19^ef^	7.25 ± 0.085^q^	42.25 ± 1.69^ef^
11	*Ocimum xcitriodorum*	8.43 ± 0.09^p^	6.08 ± 0.21^hi^	5.00 ± 0.026^n^	38.65 ± 1.43^hi^
12	*Cosmos caudatus*	24.60 ± 0.24^u^	21.34 ± 1.48^m^	17.52 ± 0.068^v^	94.68 ± 0.54^m^
13	*Pilea trinervia*	12.33 ± 0.09^s^	3.07 ± 0.12^ef^	3.54 ± 0.030^l^	40.38 ± 0.94^ef^
14	*Centella asiatica*	4.95 ± 0.03^j^	7.04 ± 0.06^j^	5.00 ± 0.027^n^	43.42 ± 1.03^j^
15	*Polyscias fruticosum*	10.29 ± 0.19^r^	21.40 ± 0.06^m^	6.49 ± 0.017^p^	94.86 ± 0.04^m^
16	*Pluchea indica*	2.83 ± 0.02^g^	37.99 ± 1.39^p^	5.62 ± 0.012^o^	93.58 ± 0.27^p^
17	*Anacardium occidentale*	5.74 ± 0.04^l^	22.72 ± 0.02^n^	7.50 ± 0.020^r^	95.11 ± 0.03^n^
18	*Cyclea barbata*	3.09 ± 0.07^h^	2.52 ± 0.22^ef^	3.35 ± 0.012^j^	23.79 ± 1.75^ef^
19	*Apium graveolens*	3.48 ± 0.01^i^	3.36 ± 0.22^f^	3.40 ± 0.011^k^	52.27 ± 1.59^f^
20	*Syzygium polyanthum*	5.58 ± 0.10^l^	25.37 ± 0.92^o^	4.54 ± 0.004^m^	93.66 ± 1.18^o^
21	*Pandanus amaryllifolius*	2.64 ± 0.03^f^	2.99 ± 0.14^ef^	1.12 ± 0.006^b^	35.99 ± 1.18^ef^
22	*Allium schoenoprasum*	1.25 ± 0.09^b^	0.08 ± 0.03^a^	1.07 ± 0.008^a^	16.69 ± 0.66^a^
23	*Allium fistulosum*	3.12 ± 0.05^h^	0.36 ± 0.02^ab^	1.24 ± 0.008^c^	25.44 ± 0.99^ab^
24	*Curcuma mangga*	6.95 ± 0.24^o^	0.85 ± 0.01^abc^	2.44 ± 0.018^g^	35.66 ± 1.93^abc^
25	*Kaempferia galanga*	0.82 ± 0.01^a^	1.53 ± 0.02^cd^	1.10 ± 0.015^ab^	22.15 ± 0.83^cd^

Data is the mean ± SD of three determinations; identical letters in the same column indicate no significant difference according to Duncan multiple comparison tests at *p* < 0.05. ^∗^Values of phenolic content and DPPH free radical scavenging activity refer to Syabana et al. [16] and Yuliana et al. [17].

## Data Availability

The data used to support the findings of this study are available from the corresponding author upon request.
